# Modeling and Analysis of a 2-DOF Spherical Parallel Manipulator

**DOI:** 10.3390/s16091485

**Published:** 2016-09-13

**Authors:** Xuechao Duan, Yongzhi Yang, Bi Cheng

**Affiliations:** 1 Key Laboratory of Electronic Equipment Structure Design, Ministry of Education of China, Xidian University, Xi’an 710126, China; yzyang@stu.xidian.edu.cn (Y.Y.); chengbi@stu.xidian.edu.cn (B.C.); 2 Collaborative Innovation Center of Information Sensing and Understanding, Xidian University, Xi’an 710126, China

**Keywords:** spherical parallel manipulator, kinematics, virtual work principle, dynamics

## Abstract

The kinematics of a two rotational degrees-of-freedom (DOF) spherical parallel manipulator (SPM) is developed based on the coordinate transformation approach and the cosine rule of a trihedral angle. The angular displacement, angular velocity, and angular acceleration between the actuators and end-effector are thus determined. Moreover, the dynamic model of the 2-DOF SPM is established by using the virtual work principle and the first-order influence coefficient matrix of the manipulator. Eventually, a typical motion plan and simulations are carried out, and the actuating torque needed for these motions are worked out by employing the derived inverse dynamic equations. In addition, an analysis of the mechanical characteristics of the parallel manipulator is made. This study lays a solid base for the control of the 2-DOF SPM, and also provides the possibility of using this kind of spherical manipulator as a 2-DOF orientation, angular velocity, or even torque sensor.

## 1. Introduction

Compared with traditional serial manipulators, the parallel manipulator is a more promising branch of robotic mechanism which has the distinct advantages of high stiffness, high precision, large load-weight ratio, high speed, and high acceleration. These advantages lead to its accordance with the trends of modern electrical and mechanical equipment. Therefore, the parallel manipulator has been a popular topic in manufacturing for decades [1,2,3,4]. With the development of robotics and automation engineering, spherical parallel mechanisms of various forms have attracted wide attention, and have found successful applications in industrial fields. In addition to the general characteristics of the parallel manipulator, the spherical parallel manipulator has the special advantages of simple structure, large workspace, interference-free of limbs, straightforward kinematics computation, reliable control, and so on. As early as 1931, Gwinnett proposed an amusement instrument based on a spherical parallel mechanism [5]. Cox developed a 3-RRR (R indicates the revolute joint) spherical 3-degrees-of-freedom (DOF) parallel mechanism [6] which is characterized by the free rotation of the mobile platform around the intersections of the revolute joint axes. Cox and Tesar proposed a 3-DOF parallel robotic shoulder module [7] which is considered as a typical spherical parallel manipulator with triangular platforms and three identical chains, each of which consisting of three revolute joints. The center of rotation of this mechanism is located at the intersection of the revolute joint axes. Asada and Granito proposed a spherical parallel manipulator with three collinear actuators [8] which improves the symmetry and convenience of the assembly of the mechanism. Gosselin and Angeles proposed a spherical 3-DOF parallel manipulator with three coplanar actuators [9] wherein the revolute joint axes of the base and mobile platforms intersect at one point. This special structure results in its preferable symmetry in both kinematics and dynamics. Moreover, Kim and Tesar implemented a force-reflecting manual controller by using this type of spherical 3-DOF parallel manipulator [10]. Gosselin et al. developed an agile eye for the orientation of an ultra-high speed camera by using a spherical 3-DOF parallel manipulator [11,12,13]. Birglen and Gosselin further developed this mechanism as a haptic device [14]. Gallardo and Rico et al. employed this mechanism in a transmission system [15]. 

Compared with 3-DOF spherical mechanisms, the study of 2-DOF spherical mechanisms is still in the preliminary stage. Zhang and Li et al. proposed a spherical 2-DOF 5R parallel manipulator based on spherical trigonometry [16] which can be used as the positioning device of a point on a spherical surface. The dynamics of the spherical 5R parallel mechanism is investigated based on the Lagrange equation [17]. Kong presented a novel 2-DOF 3–4R spherical parallel mechanism [18], but the structure of the mechanism is rather complicated and thus decreases its practicality in engineering. According to the geometrical constraint method, Xu and Chen et al. conducted motion decoupling analysis of a kind of 2R parallel mechanism with two continuous rotational axes [19]. Hu and Huang proposed a family of two-rotation and one-translation parallel manipulators with intersecting rotational axes which have identical kinematics, although they are different in structure arrangement [20]. 

The motivation of this paper is to propose a two-rotational DOF spherical parallel mechanism (SPM) based on the theory of spherical parallel mechanisms, which is eligible for vibration isolation and 2-DOF precision manipulation of all types. Considering implementation, a spherical joint is avoided in the design. The kinematics and dynamics of this mechanism will be dealt with. Moreover, according to the invertible property of a mechanism, one can swap its input and output terminals. Therefore, the miniaturization of this mechanism can be used as 2-DOF orientation and force/torque sensors by replacing the servo actuator with optical encoders and strain load cells, respectively. The advantage of this type of sensor lies in its extra load capacity; for instance, it will be able to carry a heavy instrument and measure its pitch and yaw angles. Therefore, there is no need to design a special mount when employing this kind of sensor.

We begin this paper with an introduction and a brief review of the SPMs. In Section 2, the description of the spherical 2-DOF parallel manipulator is elaborated. Then the kinematics are developed in Section 3. In Section 4, by ignoring the friction of the joints, the virtual work principle is employed to establish a dynamic model of the parallel manipulator. The motion plan of typical trajectories and simulation of the inverse dynamics are carried out in Section 5, providing a theoretical basis for the control of the manipulator. Finally, some meaningful conclusions and future work are presented in Section 6.

## 2. Description of the Spherical Parallel Manipulator

**Figure 1:**
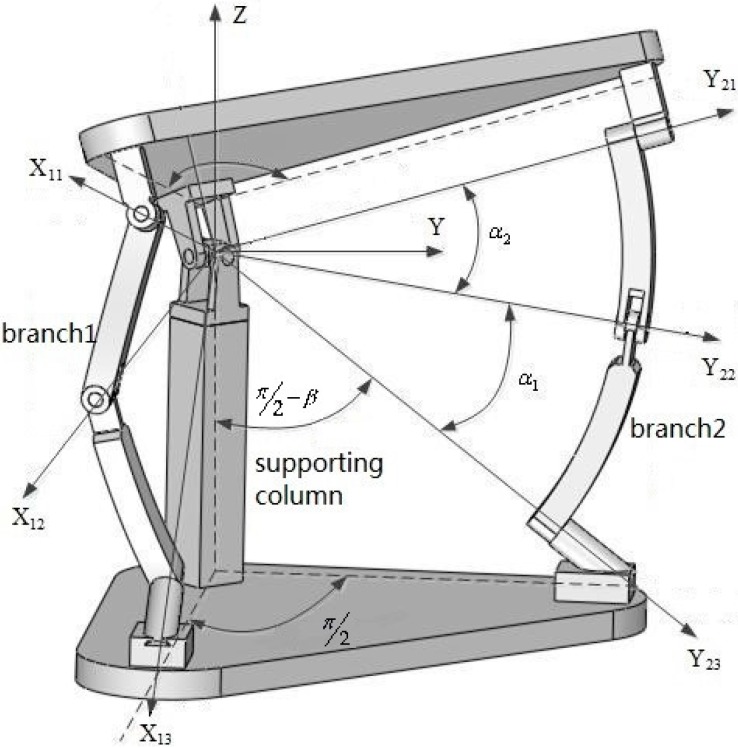
Structure of the 2-degrees of freedom (DOF) spherical parallel mechanism (SPM).

As shown in Figure 1, the lower triangular platform of the 2-DOF SPM is the base, and the upper platform is the end-effector (more generally called the mobile platform), which has only two rotational degrees of freedom. The mobile platform and the base are connected by two active branches and one vertical supporting column. All the joints between links in the two branches of the manipulator are revolute. The lower end of the supporting column is fixedly mounted to the base, and its upper end is hinged with the mobile platform with a universal joint. According to the conventions of the spherical mechanism, when putting into operation, all points on the branch linkages move on a spherical surface whose center is a specified point. Additionally, all the axes of the revolute joints intersect at a center point called the kinematic center.

The degrees of freedom of this manipulator can be verified according to the revised Kutzbach–Grubler formula [3] *M* = *d* (*n* − *g* − 1) +$\sum_{i=1}^{g}f_{i}$ + *v* − *ζ*, where *d* = 3 is the number of common constraints for the spherical mechanism, *n* is the number of parts, *g* is the number of joints, *f_i_* is the degrees of freedom of the *i*th joint, *v* is the number of redundant constraints, and *ζ* is the isolated degree of freedom. In this research, *n* = 6, and the number of joints is *g* = 7. For the universal joint, $f_{1}=2$, for the other six revolute joints, $f_{i}=1$. There is no redundant constraint and isolated degree of freedom, so v=0,ζ=0 in this research. Thus, the degrees of freedom of this mechanism are M=2.

**Figure 2:**
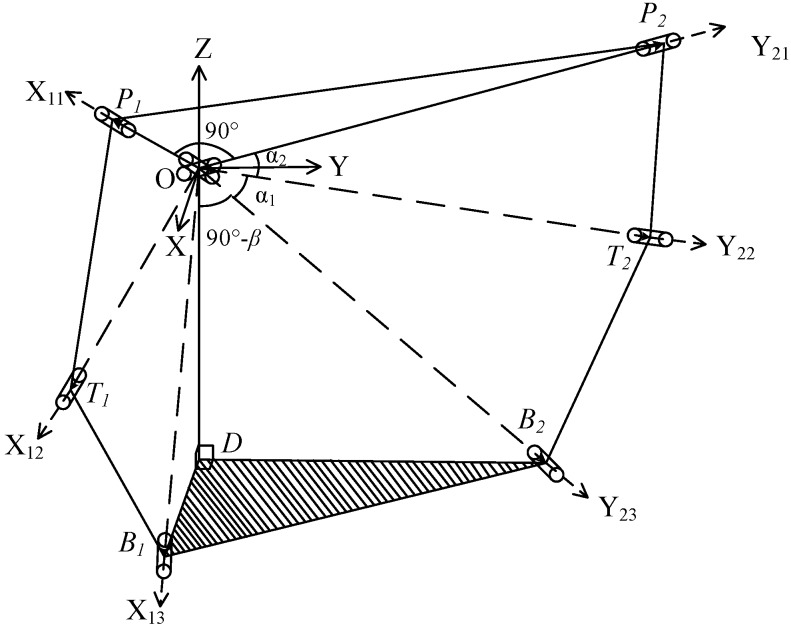
Schematic diagram of the coordinate system.

In order to describe this 2-DOF SPM, a global coordinate system *O-XYZ* and the local coordinate systems *O-X_ij_Y_ij_Z_ij_* were established, as shown in Figure 2. The subscripts *i* and *j* represent the *j*th revolute joint of the *i*th branch, respectively, where *i* = 1, 2, and *j* = 1, 2, 3. The coordinate conventions are as follows. The center point *O* of the universal joint is the origin of each coordinate system. The projection of *X* axes on the base are same direction coincident with vector $\vec{DB_{1}}$, and those of the *Y* axes are with $\vec{DB_{2}}$; thus, *Z* axes can be determined according to the right hand rule. $P_{1}$, $T_{1}$, $B_{1}$, $P_{2}$, $T_{2}$ and $B_{2}$ represent the position vectors of center point of each revolute joint in the global coordinate system. In branch 1, the axis *X*_1*j*_ (*j* = 1, 2, 3) of the local coordinate system constantly points to the center of the revolute joint from origin *O*, and *Y*_11_ coincides with *Y*_21_, *Y*_21_, and $T_{1}\times B_{1}$ are in the same direction, *Y*_13_ is in the same direction with the *Y* axis of the global coordinate system, and *Z*_1*j*_ (*j* = 1, 2, 3) can be determined by the right hand rule. In branch 2, the *Y*_2*j*_ (*j* = 1, 2, 3) of the local coordinate system constantly points to the center of the revolute joint from the origin *O*, *X*_21_ coincides with *X*_11_, *X*_22_ and $B_{2}\times T_{2}$ are in the same direction, *X*_23_ is in the same direction with the *X* axis of the global coordinate system, and *Z*_1*j*_ (*j* = 1, 2, 3) can be determined by the right hand rule. *O-X*_11_*Y*_11_*Z*_11_ coincides with *O-X*_21_*Y*_21_*Z*_21_. The position vectors of the six revolute joints in their local coordinate systems are presented, respectively, as follows:

(1) $$\begin{array}{l} p_{1}={\left(R,\;0,\;0\right)}^{T},p_{2}={\left(0,\;R,\;0\right)}^{T},b_{1}={\left(R,\;0,\;0\right)}^{T}\\ t_{1}={\left(R,\;0,\;0\right)}^{T},t_{2}={\left(0,\;R,\;0\right)}^{T},b_{2}={\left(0,\;R,\;0\right)}^{T} \end{array}$$

## 3. Kinematics 

### 3.1. Representation of the Joints

Due to the mechanical constraint of the universal joint, the mobile platform practically has only two degrees of freedom, including roll angle $\phi_{x}$ and pitch angle $\phi_{y}$. Based on the knowledge of robotics [3,21], the coordinate transformation matrices of the mobile platform in Euler angle form are:

(2) $$R_{y}(\phi_{y})=\left[\begin{array}{ccc} \cos\phi_{y} & 0 & \sin\phi_{y}\\ 0 & 1 & 0\\ -\sin\phi_{y} & 0 & \cos\phi_{y} \end{array}\right]$$

(3) $$R_{x}(\phi_{x})=\left[\begin{array}{ccc} 1 & 0 & 0\\ 0 & \cos\phi_{x} & -\sin\phi_{x}\\ 0 & \sin\phi_{x} & \cos\phi_{x} \end{array}\right]$$

where $\phi_{x}$ and $\phi_{y}$ are roll and pitch angles of the mobile platform, respectively.

In order to work out the kinematics of the 2-DOF SPM, this paper conducts a straightforward deduction. On one hand, once the mobile platform is assumed to be in a given orientation, the representation of the position of related points such as *P*_1_, *P*_2_, *T*_1_ and *T*_2_ can be obtained. On the other hand, the angular displacement of actuators will also lead to the representation of *T*_1_ and *T*_2_*.* So, the kinematic equations can be formulated according to the equivalence of *T*_1_ and *T*_2_. The details are as follows.

Assume that the original state of the 2-DOF SPM is that the local coordinate system *O-X*_11_*Y*_11_*Z*_11_ coincides with the global coordinate system *O-XYZ*. For the first operation, let the local coordinate rotate around the *Y* axis by $\phi_{y}$ to get a new coordinate system. Then, for the second operation, let the new coordinate rotate around the *X* axis by $\phi_{x}$ to get the final coordinate system. So, the resulting vectors are:

(4) $$P_{1}=R_{y}(\phi_{y})R_{x}(\phi_{x})p_{1}=R{\left(\cos\phi_{y},\;0,\;-\sin\phi_{y}\right)}^{T}$$

(5) $$P_{2}=R_{y}(\phi_{y})R_{x}(\phi_{x})p_{2}=R{\left(\sin\phi_{y}\sin\phi_{x},\;\cos\phi_{x},\;\cos\phi_{y}\sin\phi_{x}\right)}^{T}$$

Since the lower links are directly connected to the actuators, their position vectors can be represented with the input angular displacement *θ*_1_, *θ*_2_.

(6) $$T_{1}=R_{y}\left(\beta \right)R_{x}\left(\theta_{1}\right)R_{y}\left(-\alpha_{1}\right)t_{1}=R\left(\begin{array}{c} \cos\beta \cos\alpha_{1}+\sin\beta \cos\theta_{1}\sin\alpha_{1}\\ -\sin\theta_{1}\sin\alpha_{1}\\ -\sin\beta \cos\alpha_{1}+\cos\beta \cos\theta_{1}\sin\alpha_{1} \end{array}\right)$$

(7) $$T_{2}=R_{x}\left(-\beta \right)R_{y}\left(-\theta_{2}\right)R_{x}\left(\alpha_{1}\right)t_{2}=R\left(\begin{array}{c} -\sin\theta_{2}\sin\alpha_{1}\\ \cos\beta \cos\alpha_{1}+\sin\beta \cos\theta_{2}\sin\alpha_{1}\\ -\sin\beta \cos\alpha_{1}+\cos\beta \cos\theta_{2}\sin\alpha_{1} \end{array}\right)$$

Besides, the actuator locations can be described in global coordinates as,

(8) $$B_{1}=R_{y}(\beta )b_{1}=R{\left(\cos\beta ,\;0,\;-\sin\beta \right)}^{T}$$

(9) $$B_{2}=R_{x}(-\beta )b_{2}=R{\left(0,\;\cos\beta ,\;-\sin\beta \right)}^{T}$$

where *α*_1_, *β* are structural parameters as shown in Figure 1. *R* is the radius of the sphere of the 2-DOF SPM.

### 3.2. Inverse Kinematics

The inverse kinematics of a manipulator calculate the motion of actuators given the orientation of the mobile platform, which is the theoretical base for the control of the manipulator.

In branch 1, the angle between $P_{1}$ and $B_{1}$ is denoted as $\alpha_{31}$, thus

(10) $$\cos\alpha_{31}=\frac{P_{1}\cdot B_{1}}{\left|P_{1}\right|\left|B_{1}\right|}=\cos\phi_{y}\cos\beta +\sin\phi_{y}\sin\beta$$

The planes related to angles $\alpha_{31}$, $\alpha_{2}$, and $\alpha_{1}$ intersect at point *O*. The angle between the planes of $\alpha_{31}$ and $\alpha_{1}$ is denoted as $\theta_{11}$. According to cosine rule of a trihedral angle, the geometric Equation (11) is obtained.

(11) $$\cos\theta_{11}=\frac{\cos\alpha_{2}-\cos\alpha_{1}\cos\alpha_{31}}{\sin\alpha_{1}\sin\alpha_{31}}$$

The angle of the plane of $\alpha_{31}$ and *O-XZ* is set to $\theta_{12}$. According to Equation (4), $P_{1}$ and $B_{1}$ are in the plane of *O-XZ*. Thus $\theta_{12}=0$ holds. The input angle of the actuator in branch 1 is $\theta_{1}=\theta_{11}+\theta_{12}=\theta_{11}$.

In branch 2, by applying the same principle, one can obtain

(12) $$\cos\alpha_{32}=\frac{P_{2}\cdot B_{2}}{\left|P_{2}\right|\left|B_{2}\right|}=\cos\phi_{x}\cos\beta -\cos\phi_{y}\sin\phi_{x}\sin\beta$$

and

(13) $$\cos\theta_{21}=\frac{\cos\alpha_{2}-\cos\alpha_{1}\cos\alpha_{32}}{\sin\alpha_{1}\sin\alpha_{32}}$$

According to Equation (5), neither $P_{2}$ nor $B_{2}$ is in the plane of *O-YZ*. Thus, $\theta_{22}\neq 0$. In order to obtain $\theta_{22}$ between *O-P*_2_*B*_2_ and *O-YZ*, the intermediate vector $\vec{j}={\left(0,\;R,\;0\right)}^{T}$ is introduced.

(14) $$\cos\theta_{22}=\frac{\left(B_{2}\times P_{2}\right)\cdot \left(B_{2}\times \vec{j}\right)}{\left|B_{2}\times P_{2}\right|\left|B_{2}\times \vec{j}\right|}=-\sin\beta \left(\cos\beta \cos\phi_{y}\sin\phi_{x}-\sin\beta \cos\phi_{x}\right)$$

Therefore, the angle of actuator 2 is worked out as $\theta_{2}=\theta_{21}+\theta_{22}$.

### 3.3. Forward Kinematics

The forward kinematics of this 2-DOF SPM calculate the orientation of the mobile platform, provided the angular displacement of the two actuators. This procedure is of great importance for the orientation sensor application of this SPM. More generally, the semi-closed loop control of the parallel mechanism also needs its forward kinematics so that the orientation can be obtained by reading out the feedback values of the actuators and a further computation.

The angle between vectors $T_{1}$ and $P_{1}$ is constantly equal to $\alpha_{2}$, which can be expressed by

(15) $$\frac{T_{1}\cdot P_{1}}{\left|T_{1}\right|\left|P_{1}\right|}=\cos\alpha_{2}$$

By solving Equation (15), the following solution of $\phi_{y}$ can be obtained

(16) $$\phi_{y}=\arcsin\frac{c_{1}}{\sqrt{a_{1}^{2}+b_{1}^{2}}}-\arctan\frac{b_{1}}{a_{1}}$$

where $a_{1}=\sin\beta \cos\alpha_{1}-\cos\beta \cos\theta_{1}\sin\alpha_{1},\;b_{1}=\cos\beta \cos\alpha_{1}+\sin\beta \cos\theta_{1}\sin\alpha_{1},\;c_{1}=\cos\alpha_{2}$.

Similarly, the angle between $T_{2}$ and $P_{2}$ is constantly equal to $\alpha_{2}$; thus, one can obtain

(17) $$\frac{T_{2}\cdot P_{2}}{\left|T_{2}\right|\left|P_{2}\right|}=\cos\alpha_{2}$$

By calculating Equation (17), the following parameter can be obtained

(18) $$\phi_{x}=\arccos\frac{c_{2}}{\sqrt{a_{2}^{2}+b_{2}^{2}}}+\arctan\frac{a_{2}}{b_{2}}$$

where $a_{2}=-\sin\theta_{2}\sin\alpha_{1}\sin\phi_{y}+\left(\cos\beta \cos\theta_{2}\sin\alpha_{1}-\sin\beta \cos\alpha_{1}\right)\cos\phi_{y},\;b_{2}=\cos\beta \cos\alpha_{1}+\sin\beta \cos\theta_{2}\sin\alpha_{1},\;c_{2}=\cos\alpha_{2}$.

From the solutions to the forward kinematics mentioned above, one can infer that the rotation of the mobile platform around the *Y* axis is only dependent on the motion of actuator 1. Compared with this conclusion, the rotation of the mobile platform around *X* axis is dependent on the motion of both actuators.

### 3.4. Analysis of Velocity and Acceleration

The velocity and acceleration analysis plays a key role in the mechanical design, controller design, and hardware configuration. Let $\phi ={\left(\phi_{y},\;\phi_{x}\right)}^{T}$,$\dot{\phi }={\left({\dot{\phi }}_{y},\;{\dot{\phi }}_{x}\right)}^{T}$, and $\ddot{\phi }={\left({\ddot{\phi }}_{y},\;{\ddot{\phi }}_{x}\right)}^{T}$ be the orientation, rotation velocity, and rotation acceleration vectors of the mobile platform, respectively. Let $\theta ={\left(\theta_{1},\;\theta_{2}\right)}^{T}$,$\dot{\theta }={\left({\dot{\theta }}_{1},\;{\dot{\theta }}_{2}\right)}^{T}$, and $\ddot{\theta }={\left({\ddot{\theta }}_{1},\;{\ddot{\theta }}_{2}\right)}^{T}$ be the angular displacement, angular velocity, and angular acceleration vectors of the actuators, respectively. This section will deal with the input–output relation of the velocity and acceleration of the 2-DOF SPM.

By differentiating Equations (16) and (18) with respect to time, Equation (19) can be obtained in the following form

(19) $$A\dot{\phi }+B\dot{\theta }=0$$

where, $A=\left[\begin{array}{cc} f_{11} & 0\\ f_{21} & f_{22} \end{array}\right]$, $B=\left[\begin{array}{cc} l_{11} & 0\\ 0 & l_{22} \end{array}\right]$.

$$\begin{array}{l} f_{11}=\left(\cos\alpha_{1}\sin\beta -\cos\beta \cos\theta_{1}\sin\alpha_{1}\right)\cos\phi_{y}-\left(\cos\alpha_{1}\cos\beta +\cos\theta_{1}\sin\alpha_{1}\sin\beta \right)\sin\phi_{y}\\ f_{21}=\left(\cos\alpha_{1}\sin\beta -\cos\beta \cos\theta_{2}\sin\alpha_{1}\right)\sin\phi_{y}\sin\phi_{x}-\sin\alpha_{1}\sin\theta_{2}\cos\phi_{y}\sin\phi_{x}\\ f_{22}=\cos\phi_{x}\left[\cos\phi_{y}\left(\cos\beta \cos\theta_{2}\sin\alpha_{1}-\cos\alpha_{1}\sin\beta \right)-\sin\alpha_{1}\sin\theta_{2}\sin\phi_{y}\right]-\left(\cos\alpha_{1}\cos\beta +\cos\theta_{2}\sin\alpha_{1}\sin\beta \right)\sin\phi_{x}\\ l_{11}=-\cos\phi_{y}\sin\alpha_{1}\sin\beta \sin\theta_{1}+\cos\beta \sin\alpha_{1}\sin\theta_{1}\sin\phi_{y}\\ l_{22}=-\cos\phi_{x}\sin\alpha_{1}\sin\beta \sin\theta_{2}-\cos\beta \cos\phi_{y}\sin\alpha_{1}\sin\theta_{2}\sin\phi_{x}-\cos\theta_{2}\sin\alpha_{1}\sin\phi_{x}\sin\phi_{y} \end{array}$$

Additionally, Equation (17) can be rewritten in another form:

(20) $$\dot{\phi }=\left[G_{\phi }\right]\dot{\theta }$$

where $G_{\phi }=-A^{-1}B$ is the first-order rotation influence coefficient matrix on the 2-DOF rotations of the mobile platform. One can conclude that the angular velocity of the mobile platform relates to the angular displacement and angular velocity of the actuators. 

Differentiation of Equation (19) with respect to time leads to the following differential equation

(21) $$\frac{dA}{dt}\dot{\phi }+A\ddot{\phi }+\frac{dB}{dt}\dot{\theta }+B\ddot{\theta }=0$$

Equation (21) can be rewritten in another form:

(22) $$\ddot{\phi }=-A^{-1}\left[\begin{array}{cc} \frac{\partial f_{11}}{\partial \phi^{T}}\dot{\phi }+\frac{\partial f_{11}}{\partial \theta^{T}}\dot{\theta } & 0\\ \frac{\partial f_{21}}{\partial \phi^{T}}\dot{\phi }+\frac{\partial f_{21}}{\partial \theta^{T}}\dot{\theta } & \frac{\partial f_{22}}{\partial \phi^{T}}\dot{\phi }+\frac{\partial f_{22}}{\partial \theta^{T}}\dot{\theta } \end{array}\right]\dot{\phi }-A^{-1}\left[\begin{array}{cc} \frac{\partial l_{11}}{\partial \phi^{T}}\dot{\phi }+\frac{\partial l_{11}}{\partial \theta^{T}}{\dot{\theta }}^{T} & 0\\ 0 & \frac{\partial l_{22}}{\partial \phi^{T}}\dot{\phi }+\frac{\partial l_{22}}{\partial \theta^{T}}{\dot{\theta }}^{T} \end{array}\right]\dot{\theta }-A^{-1}B\ddot{\theta }$$

where *A* and *B* are matrices as shown in Equation (19). From this result, one can likewise notice that the angular acceleration of the mobile platform relates to the input angular velocity, input angular acceleration, and the orientation and structural parameters of the manipulator.

## 4. The Dynamics Model Based on the Virtual Work Principle

The investigation of dynamics is dealing with the quantitative relation between the motion of the mobile platform and the input torques provided by the actuating joints. Furthermore, calculating the force or torque of each actuator on the active joint for the desired trajectory of the mobile platform is called inverse dynamics. In this research, since the mobile platform has thirty times greater mass than that of the four linkages, the mass of the four linkages is thus negligible for simplicity.

The inertial matrix tenor of the mobile platform is

(23) $$\left[I_{c}\right]=\left[\begin{array}{cc} I_{xx} & 0\\ 0 & I_{yy} \end{array}\right]$$

where Ixx=Iyy=14mr2, *m* is the mass of the mobile platform, and *r* is the radius of the mobile platform.

**Figure 3:**
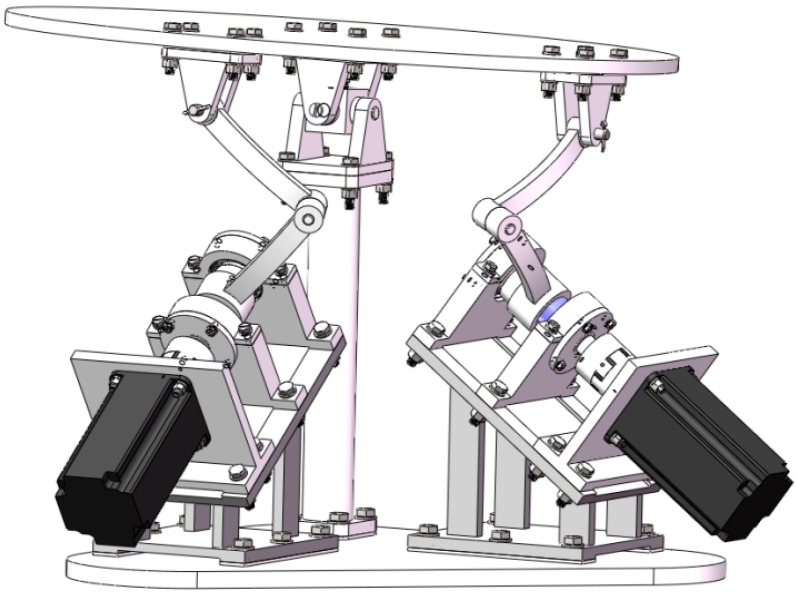
Virtual prototype.

It is to be noted that for this 2-DOF SPM prototype, the center of gravity lies in the point above the center of the universal joint by a distance of *h*, as shown in Figure 3. Therefore, the position vector of the center of gravity of the mobile platform is

(24) $$p_{cog}=h{\left(\sin\phi_{y}\cos\phi_{x},\;-\sin\phi_{y},\;\cos\phi_{y}\cos\phi_{x}\right)}^{T}$$

where $\phi_{x}$ and $\phi_{y}$ are roll and pitch angles of the mobile platform, respectively. 

The differentiation of ***p**_cog_* with respect to time results in:

(25) $$v=h\left[\begin{array}{cc} \cos\phi_{y}\cos\phi_{x} & \sin\phi_{y}\sin\phi_{x}\\ -\cos\phi_{y} & 0\\ -\sin\phi_{y}\cos\phi_{x} & \cos\phi_{y}\sin\phi_{x} \end{array}\right]\dot{\phi }$$

Equation (25) can be rewritten in the following form:

(26) $$v=\left[G_{v}\right]\dot{\phi }$$

where $\left[G_{v}\right]$ is the first-order translation influence coefficient matrix on the motion of the center of gravity of the mobile platform. The translation here especially indicates the translation of the center of gravity of the mobile platform.

By differentiating Equation (26) with respect to time, one can obtain:

(27) $$a=h\left[\begin{array}{cc} {\dot{\phi }}^{T}\left(\begin{array}{c} -\cos\phi_{x}\sin\phi_{y}\\ -\sin\phi_{x}\cos\phi_{y} \end{array}\right) & {\dot{\phi }}^{T}\left(\begin{array}{c} \sin\phi_{x}\cos\phi_{y}\\ \cos\phi_{x}\sin\phi_{y} \end{array}\right)\\ {\dot{\phi }}^{T}\left(\begin{array}{c} \sin\phi_{y}\\ 0 \end{array}\right) & 0\\ {\dot{\phi }}^{T}\left(\begin{array}{c} -\cos\phi_{x}\cos\phi_{y}\\ \sin\phi_{x}\sin\phi_{y} \end{array}\right) & {\dot{\phi }}^{T}\left(\begin{array}{c} -\sin\phi_{x}\sin\phi_{y}\\ \cos\phi_{x}\cos\phi_{y} \end{array}\right) \end{array}\right]\dot{\phi }+\left[G_{v}\right]\ddot{\phi }$$

Based on the virtual work principle, the dynamic equation ${\dot{\theta }}^{T}\tau ={\dot{\phi }}^{T}\left[I_{c}\right]\ddot{\phi }+v^{T}ma+v^{T}mg$ is obtained. By substituting Equations (26) and (27) into this equation, the inverse dynamic equation of the 2-DOF SPM can be obtained as follows,

(28) $$\tau ={\left[G_{\phi }\right]}^{T}\left[I_{c}\right]\ddot{\phi }+m{\left[G_{\phi }\right]}^{T}{\left[G_{v}\right]}^{T}\left(a+g\right)$$

where $g={\left(0,\;0,\;-g_{0}\right)}^{T}$ is the gravitational acceleration vector. $\left[G_{\phi }\right]$ and $\left[G_{v}\right]$ are the two influence matrices of the input on the rotation and motion of the center of gravity of the mobile platform, respectively. *m* and $\left[I_{c}\right]$ are the mass and inertial tensor of the mobile platform, respectively. ***a*** is the intermediate motion parameter as defined in Equation (27). This dynamic equation describes the relation of input torque and the resulting motion of the mobile platform.

## 5. Numerical Simulation

To validate the theoretical results derived above, the motion planning, inverse, forward kinematic simulation, and inverse dynamics simulation were conducted. The parameters of the 2-DOF SPM for this simulation are listed in Table 1.

For the first typical trajectory motion following simulation, a linear orientation variation for 2 s is designed in which the orientation of the mobile platform can be described by Equation (29) during time 0≤t≤2 s.

(29) $$\left\{ \begin{array}{l} \phi_{y}(t)=5{}^{\circ}{\left(t-2\right)}^{2}-10{}^{\circ}\\ \phi_{x}(t)=-5{}^{\circ}{\left(t-2\right)}^{2}+10{}^{\circ} \end{array}\right.$$

**Figure 4:**
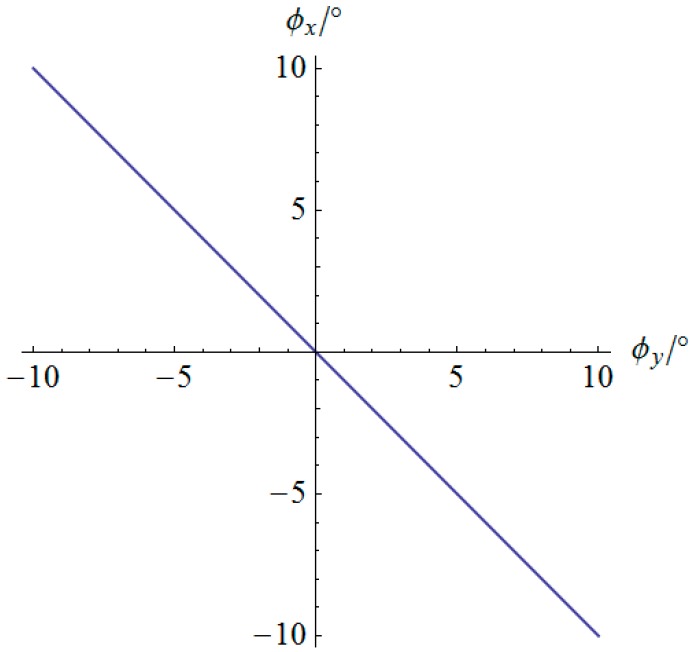
Angular displacement of the mobile platform.

**Figure 5:**
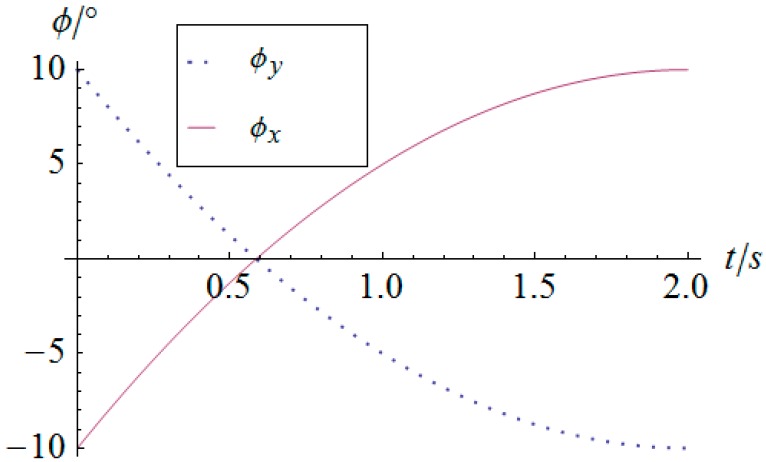
Angular displacement vs. time of the actuators.

**Figure 6:**
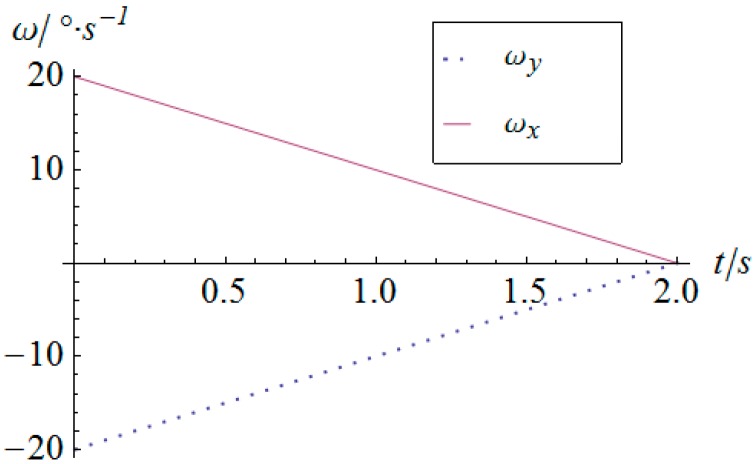
Angular velocity vs. time of the mobile platform.

**Figure 7:**
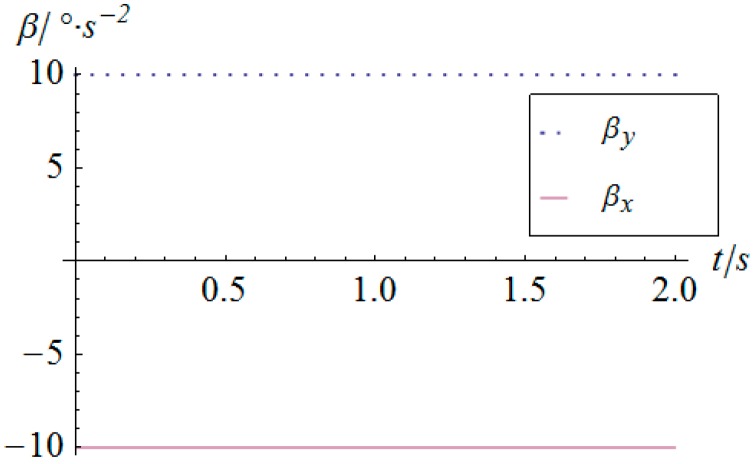
Angular acceleration vs. time of the mobile platform.

**Figure 8:**
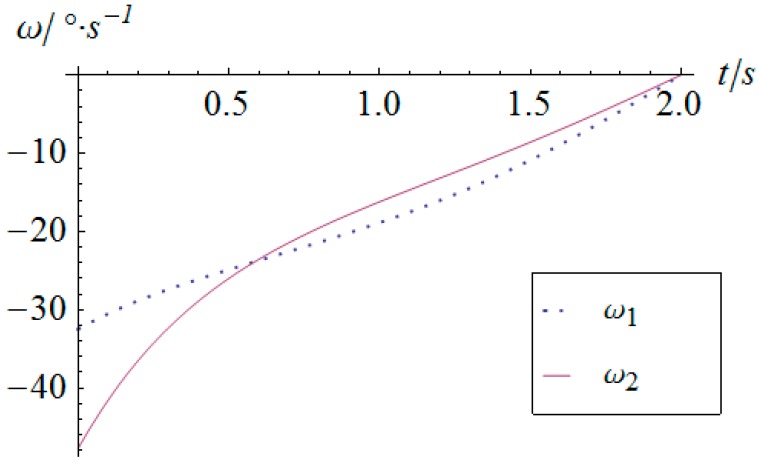
Angular velocity vs. time of the actuators.

**Figure 9:**
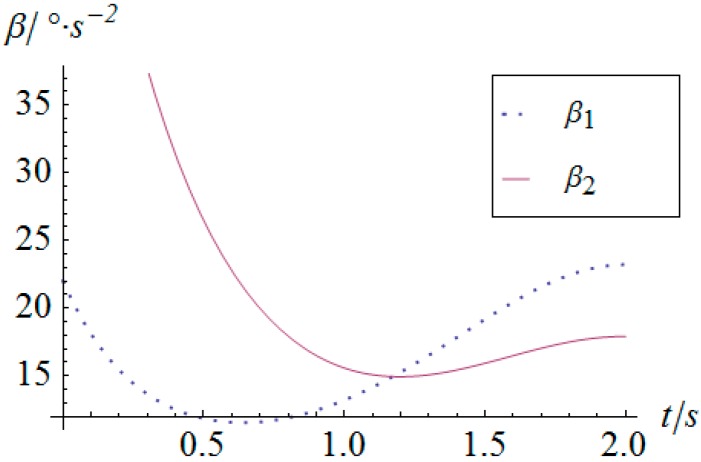
Angular acceleration vs. time of the actuators.

**Figure 10:**
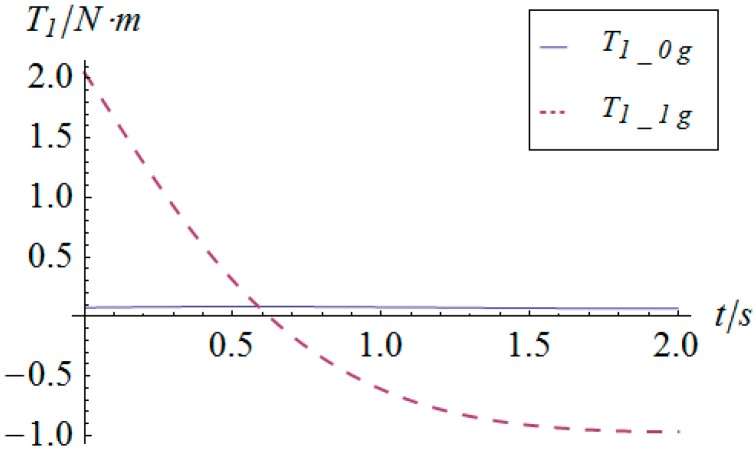
Driving torque vs. time of actuator 1.

**Figure 11:**
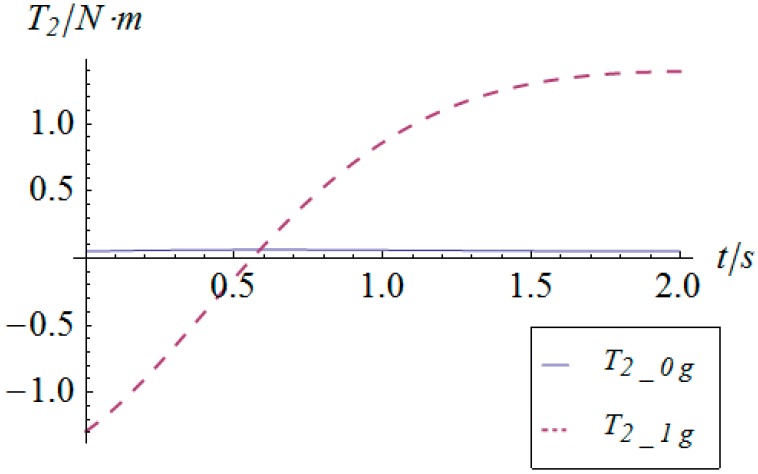
Driving torque vs. time of actuator 2.

The angular displacement of the mobile platform is shown in Figure 4. The resultant rotational motion of the mobile platform displays strict linearity. By using the inverse kinematics developed in Section 3.2, the angular displacement curves of the two actuators can be worked out, as shown in Figure 5. One can observe that the actuator displays parabolic curves as a result of the quadratic rotational motion function defined in Equation (29). Then, the angular velocity and angular acceleration of the mobile platform can be derived as shown in Figure 6 and Figure 7, respectively. The angular velocity and angular acceleration of the two actuators can be obtained as shown in Figure 8 and Figure 9, respectively. The differential relation between the velocity and acceleration and also the symmetry in two rotational degrees of freedom can be observed. Based on the dynamic equation shown in Equation (28), the driving torque curves of the actuators are shown in Figure 10 and Figure 11. In each of them, the dashed line (*T_i__*_1*g*_) and solid line (*T_i__*_0*g*_) represent the driving torque with and without gravity, respectively, from which one can notice that the linear angular acceleration of the mobile platform leads to its relatively small constant inertial force supplied by the actuators, while the gravity of the mobile platform accounts for a large proportion of the driving torque in this type of uniformly accelerated motion. 

In addition, for the typical sinusoidal trajectory motion following simulation, the orientation of the mobile platform can be described by Equation (30) during time 0≤t≤2s.

(30) $$\left\{ \begin{array}{l} \phi_{y}(t)=10{}^{\circ}\cos(\pi t)\\ \phi_{x}(t)=10{}^{\circ}\sin(\pi t) \end{array}\right.$$

**Figure 12:**
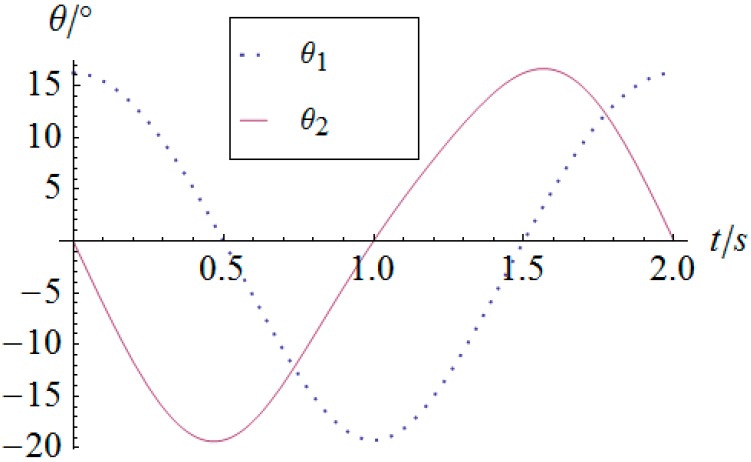
Angular displacement vs. time of the actuators.

**Figure 13:**
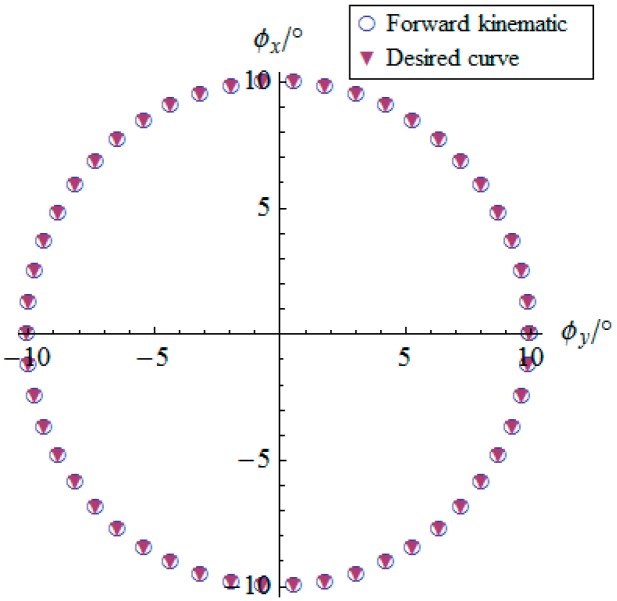
Angular displacement of the mobile platform.

By using the inverse kinematics developed in Section 3.2, the angular displacement curves of the two actuators are shown in Figure 12. One can observe that the actuator displays periodical curves slightly different from canonical sinusoidal or cosine curves. Moreover, Figure 13 illustrates the comparison between the desired orientation curve and the forward kinematic result derived from curves in Figure 12. It is clear that the inverse and forward kinematics obtained in Section 3.3 agree with each other, which validates the forward kinematics deduced in this research. As this structure contains two serial chains, the forward kinematics has the analytical form instead of iterative solutions usually appearing in parallel mechanisms of all types. It is efficient to calculate the orientation of the mobile platform once to get the angular information of the active joints. This fact is one of the distinct characteristics of the possibility of using this 2-DOF SPM as a 2-DOF orientation sensor.

**Figure 14:**
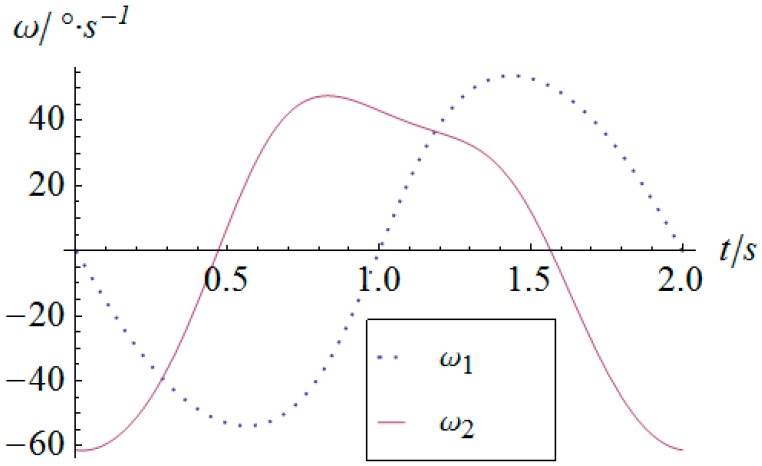
Angular velocity vs. time of the actuators.

**Figure 15:**
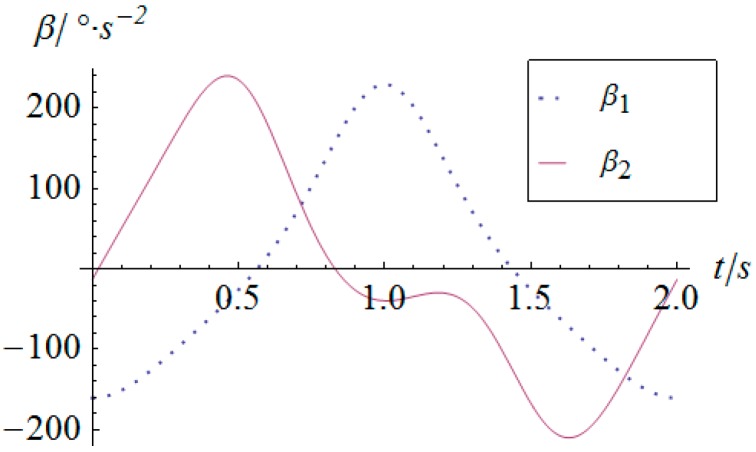
Angular acceleration vs. time of the actuators.

As for the derivatives of the angular displacement, the angular velocity and angular acceleration of the two actuators can be derived as shown in Figure 14 and Figure 15, respectively. Because of the sharp variation of the angular displacement of actuator 2 at the moment of t=0, its angular velocity is quite considerable, as shown in Figure 14. Especially for the angular acceleration, the peak value gets as much as 248 °/s^2^. This kind of rigid impulse is very harmful for heavy load applications. Therefore, in a practical motion of the SPM, this sharp jump of acceleration should be avoided or decreased by trapezoidal or high-order polynomial velocity motion planning.

**Figure 16:**
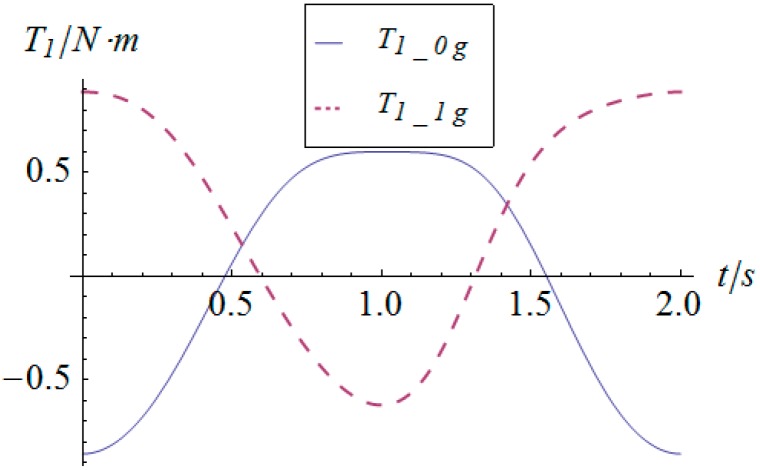
Torque vs. time of actuator 1.

**Figure 17:**
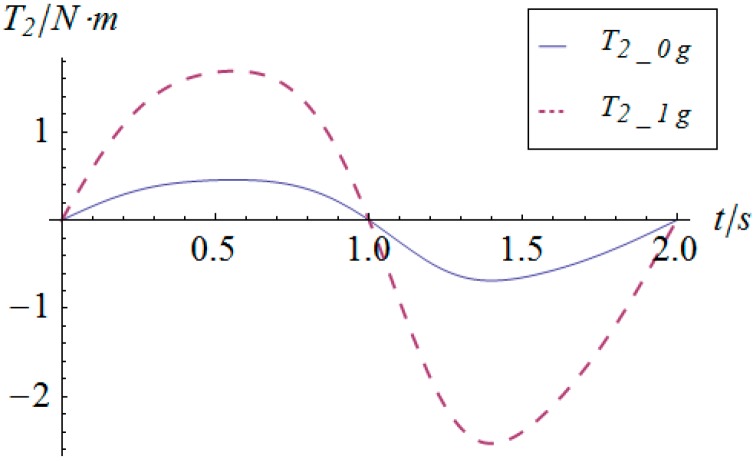
Torque vs. time of actuator 2.

Similarly, based on the dynamic equation expressed in Equation (28), the driving torque curve of the actuators in this sinusoidal trajectory tracking simulation are shown in Figure 16 and Figure 17, in which *T_i__*_1*g*_ and *T_i__*_0*g*_ represent the driving torque with and without gravity, respectively. From these figures, one can observe that the maximum torque of both actuators is less than 2.5 Nm, which satisfies the provided performance of general commercial middle and small inertia AC servo motors. As for the comparison of the driving torque under conditions with and without gravity, the conclusion for the linear acceleration case does not apply in this simulation due to the fact that the accelerations are highly time-varying and in larger magnitude.

**Figure 18:**
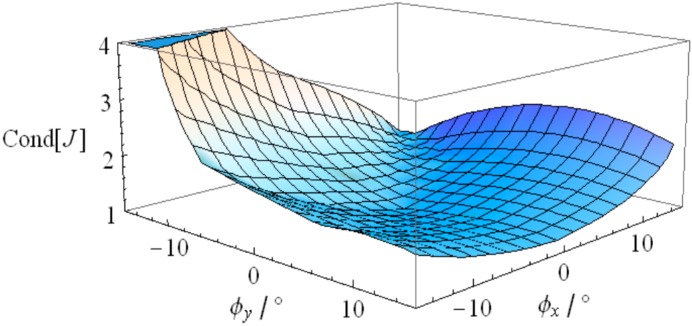
Condition number of the 2-DOF SPM.

To investigate the singularity of the 2-DOF SPM, its first-order influence coefficient matrix is considered as the Jacobian matrix *J* in this research. The mapping of condition number when the orientation of the mobile platform is an arbitrary value within the workspace is shown in Figure 18. Since the condition number lies in the rational finite interval for the orientation of the mobile platform, one can reach the conclusion that this mechanism is singularity-free in the workspace. Additionally, the condition number varies with the combination of the two rotational angles rather than keeping constant, which implies that this mechanism fails to achieve homogenous performance, especially for the error and static generalized force transmission from the active joints to the mobile platform. This is also the reason why the driving force of the actuator displays a high value for certain orientations.

**Figure 19:**
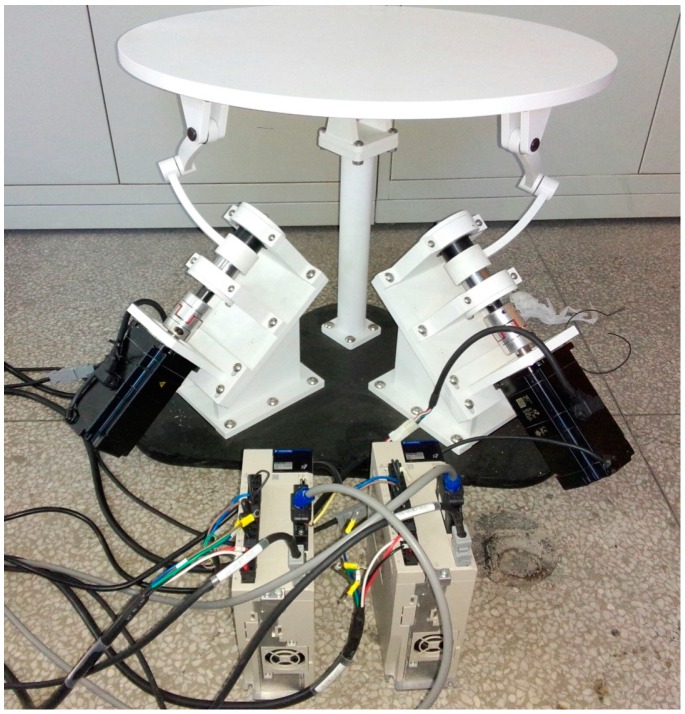
Field photo of the 2-DOF SPM prototype.

As a tentative prototype, the 2-DOF SPM as shown in Figure 19 was fabricated, the diameter of the mobile platform was 600 mm, the height of the vertical column was 378.6 mm, and the total mass was 73.67 kg. Two YASKAWA MSMA AC servo motors (SGMGV-09A) were employed as the actuators. The basic motion was achieved with the Googol CPAC motion controller as the master control unit. An elementary motion control containing two degrees of freedom was firstly carried out, validating the structural design and kinematic model. As future work, the thorough verification of the precision, workspace, and dynamic performance should be conducted.

## 6. Conclusions 

The kinematics of a 2-DOF SPM was elaborated based on the coordinate transformation approach and cosine rule of a trihedral angle. Moreover, the angular displacement, angular velocity, and angular acceleration relation between the actuators and the end-effector were determined. The dynamic model of the parallel manipulator were established by employing the virtual work principle and the first-order influence coefficient matrix of the manipulator. Finally, typical numerical simulations of the 2-DOF SPM reveal the quantitative relation between the input and output in terms of angular displacement, angular velocity, and acceleration. This preliminary result lays the foundation for better planning motion and control of the 2-DOF SPM, and also provides the fundamental verification of the possibility of using this kind of spherical mechanism as 2-DOF orientation or torque sensors.

## Figures and Tables

**Table 1:** Simulation parameters.

α_1_/°	α_2_/°	β/°	m/kg	g_0_/N·kg^−1^		r/m	h/m
25	30	36.87	27	9.8	0.3		0.026
